# The design and implementation of natural population cohort study Biobank: A multiple-center project cooperation with medical consortia in Southwest China

**DOI:** 10.3389/fpubh.2022.996169

**Published:** 2022-12-01

**Authors:** Ping Fan, Shu Zhang, Weiya Wang, Zongze Yang, Weiwei Tan, Shujun Li, Chenxing Zhu, Dan Hu, Xinran Zhou, Zixuan Tian, Yaxi Wang, Fang Liu, Wei Huang, Lei Chen

**Affiliations:** ^1^West China Biobanks and National Clinical Research Center for Geriatrics, West China Hospital, Sichuan University, Chengdu, China; ^2^Department of Pathology, West China Hospital, Sichuan University, Chengdu, China; ^3^Department of Clinical Research Management, West China Hospital, Sichuan University, Chengdu, China; ^4^West China Centre of Excellence for Pancreatitis, Institute of Integrated Traditional Chinese and Western Medicine, West China Hospital, Sichuan University, Chengdu, China; ^5^Institutes for Systems Genetics & Immunology and Inflammation, Frontiers Science Centre for Disease-Related Molecular Network, West China Hospital, Sichuan University, Chengdu, China

**Keywords:** natural population cohort study, medical consortia, Biobank, big data, health management policies

## Abstract

**Objective:**

The West China Hospital of Sichuan University collaborated with regional medical consortia in Sichuan Province to launch a natural population cohort study (NPCS) to investigate the health status of residents and collect public health data in southwest China.

**Methods:**

Up to 80,000 participants will be enrolled by the NPCS from 11 regional medical consortia over five years. Individuals are invited to visit one of 11 participating medical consortia to fill out questionnaires, receive a free health exam, and donate biospecimens upon enrolment. All participating medical facilities adhered to standard operating procedures for collecting and processing biospecimens to ensure uniformity (serum, lithium heparinized plasma, ethylene diamine tetraacetie acid plasma, and buffy coat). The Electronic Data Capture System, Picture Archiving and Communication System, Laboratory Information Management System, Biospecimen Quality Control System, Biobank Information Management System, and will be used to sort and classify clinical indices, imaging data, laboratory parameters, pre-analytical variables, and biospecimen information, respectively. All quality assurance and quality control procedures in the NPCS biobank adhered to the “DAIDS Guidelines for Good Clinical Laboratory Practice Standards”. This project will integrate high-dimensional multi-omics data, laboratory data, clinical data, questionnaire data, and environmental risk factors.

**Results:**

An estimated 2,240,000 aliquots of the sample will be stored by the end of the study. These samples are linked with comprehensively collected clinical indices, imaging data, and laboratory parameters. Big data analysis can be implemented to create predictive algorithms, explore pathogenesis mechanisms, uncover potential biomarkers, and provide information on public health.

**Conclusions:**

NPCS will provide an integrative approach to research risk factors and pathogenesis of major chronic or endemic diseases in Sichuan Province and provide key scientific evidence to support the formulation of health management policies in China.

## Introduction

China has the largest elderly population and is one of the fastest-aging countries worldwide according to the national “14th Five-Year Plan.” Moreover, according to the National Health Commission, during the “14th Five-Year” period, the degree of the aging population will further increase, and people aged ≥60 years old will account for >20% of the total population in China. Consequently, the prevalence of chronic diseases and their associated burdens have increased substantially, posing a significant public health threat to the country (1–5). To date, the Chinese government has endeavored to ensure that all citizens have equal access to high-quality healthcare. These efforts include increasing financial and infrastructure investment in the healthcare system (6), as well as publishing a series of national guidelines for the management of chronic diseases (Supplementary Table 1) such as stroke; ischemic heart disease; tracheal, bronchus, and lung cancer; chronic obstructive pulmonary disease; liver cancer, and so forth (7). Local governments and all levels of medical institutions are also mobilized to implement national health initiatives. These initiatives include enhancing the quality of training for the new and current primary health care workforce, establishing performance accountability to incentivize primary healthcare with high quality and value, combining clinical care with basic public health services, enhancing the coordination between institutions and hospitals for primary health care, and improving information systems to develop a learning and self-evolving system for primary healthcare (6).

Sichuan Province, located in southwest China, is the fifth largest province (481,400 km^2^) with a fifth-ranking population of over 83.7 million. Many factors have increased the incidence of chronic diseases in Sichuan Province, including poor living habits and changes in dietary structure, such as a high prevalence of smoking, drinking, unhealthy diet, lack of exercise, increased social pressure, and environmental impact. In addition, the increased aging population can lead to a higher number of patients with chronic diseases, such as cancer (8, 9). The major chronic diseases in Sichuan Province are tracheal, bronchus, and lung cancer; chronic obstructive pulmonary disease; lower respiratory infection; esophageal cancer; stomach cancer; colon and rectum cancer; liver cancer; cirrhosis; and other chronic liver diseases, which have a higher prevalence than the respective disease average prevalence in China. The West China Hospital of Sichuan University (WCH/SCU), the only hospital in Sichuan Province directly overseen by the Chinese Health Commission, has extensive experience in conducting multicenter studies. In recent years, prospective, large-scale cohort studies have become increasingly effective tools in epidemiology and public health (Supplementary Table 2) (10–25). Therefore, the WCH/SCU sought to establish a natural population cohort study (NPCS) that fully encompasses the region's multiethnic distribution, unique natural conditions, and lifestyle characteristics. WCH/SCU extended invitations to 11 regional consortia centers from the province that were distributed equally in terms of geography. In addition to the above-mentioned major chronic diseases, this project also aims to investigate endemic diseases (which are highly prevalent in the Sichuan population), such as Keshan disease, Kaschin-Beck disease, and skeletal fluorosis. This project will integrate high-dimensional multi-omics data, laboratory data, clinical data, questionnaire data, and environmental risk factors, such as particulate matter 2.5, passive smoking, gases, fumes, and so on. Thus, big data analysis (i.e., PheWAS) (26, 27) can be implemented to create predictive algorithms, explore pathogenesis mechanisms, uncover potential biomarkers, and provide information on public health.

This paper outlines the NPCS design; multicenter coordination and workflow; operation of Biobanks; and processes involved in the collection, handling, shipment, long-term storage, and standardized maintenance of biospecimens.

## Methods and analysis

### Design

The NPCS is a population-based multicenter cohort study conducted by the Department of Clinical Research at the WCH/SCU. This study was approved by the ethics committee of the WCH/SCU [No. (2020)145] and received a license from the Ministry of Science and Technology of China [No. (2020) CJ0150]. The Biobank is accredited by the China Human Genetic Resources Management Office as part of the West China Biobanks of the WCH/SCU [No. (2016)406 and No. (2022) BC0040]. The same informed consent form is used at all sites of the 11 medical consortia centers. In the new version of the informed consent form (updated on November 12, 2020), the statement “I voluntarily accept the survey process of this research questionnaire to be recorded and saved, and the members of the project team to be allowed to re-examine the questionnaire” was added to consolidate the quality of questionnaire results. Informed consent was obtained from all participants before they entered the NPCS.

### Selection/treatment of participants

**Figure 1:**
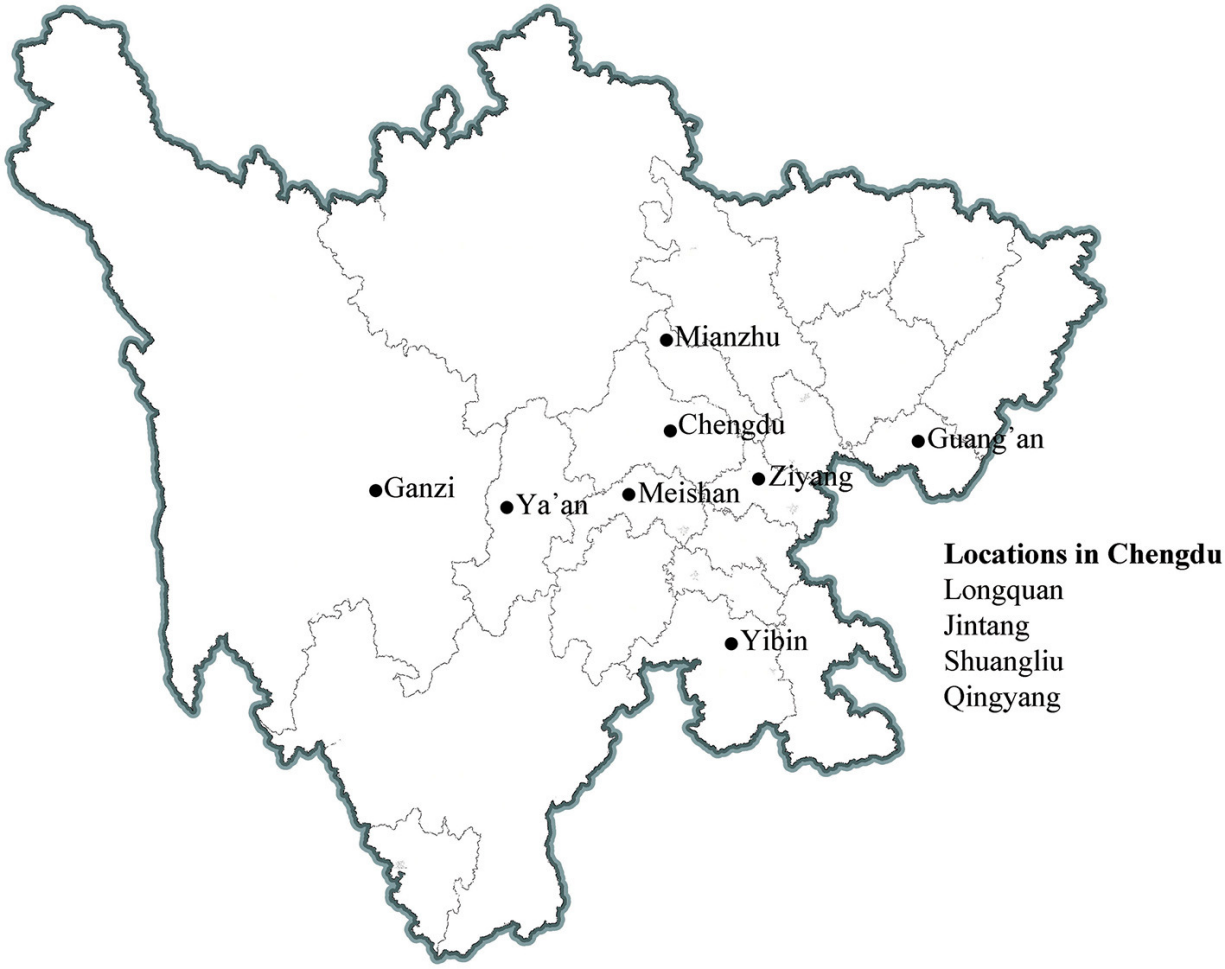
Locations of 11 survey sites in Sichuan Province.

The source population of the NPCS is from the locations of 11 participating medical consortia concerning approximately 2% of the local population (Figure 1). The cohort utilized cluster sampling (community or village) for population recruitment. Adult participants and their relatives who (1) are aged 20 years or over, (2) had local household registration or non-local household registration but lived in the area for more than 6 months were invited to participate. Subsequently, a questionnaire (Supplementary Table 3) that covers their occupation, lifestyle, symptoms and signs, medical history, and so on is filled out by the included participants. They are also invited to participate in a free comprehensive health exam that included biometry (height, weight, and waist circumference), blood pressure and pulse, and pulmonary function. Between these procedures, blood and urine samples are collected from participants when available (Table 1). Blood samples (4 mL) in ethylenediaminetetraacetic acid (EDTA; plasma) and 1.5 mL in serum separator tubes (SST; serum) are collected for measuring biological parameters related to whole blood cell counts, glucose metabolism, lipid profile, and electrolytes, as well as liver and renal function. Another blood sample of 9 mL in EDTA, 3 mL of lithium heparin (LH; plasma), and 3 mL of SST are collected for long-term storage. Urine is collected for routine testing. Blood biospecimens for long-term storage are transported and stored centrally at the West China Biobanks of the WCH/SCU for future research. Participants older than 40 years are invited to undergo a computerized tomography (CT) chest scan. The participants are followed for each 1–2 years over a 5 year period. The NPCS aims to maintain a lost of follow-up rate of ≤ 10% whenever possible. A set of standard operating procedures (SOPs) has been established to ensure high-quality implementation, and professional information management systems are used to store health information and the NPCS. Overall, from April 2020 to June 2022, approximately 40,000 participants have been included, and up to 40,000 participants will be recruited between November 2022 and February 2025.

**Table 1:** Type of biospecimen, collection priority, and volume.

**Type of biospecimen**	**Collection priority**	**Collection volume (mL)**	**Derivative**	**Usage**
EDTA	1	4	Plasma	Whole blood cell counts, glucose metabolism, lipid profile, and electrolytes test
SST	2	1.5	Serum	Liver and renal function test
Urine	3	50	/	Routine urine test
EDTA	4	9	Plasma	Long-Term storage
			Buffy coat	Long-Term storage
LH	5	3	Plasma	Long-Term storage
SST	6	3	Serum	Long-Term storage

EDTA, ethylene diamine tetraacetic acid; LH, lithium heparin; SST, serum separator tube.

### Task assignment with local medical consortia

**Figure 2:**
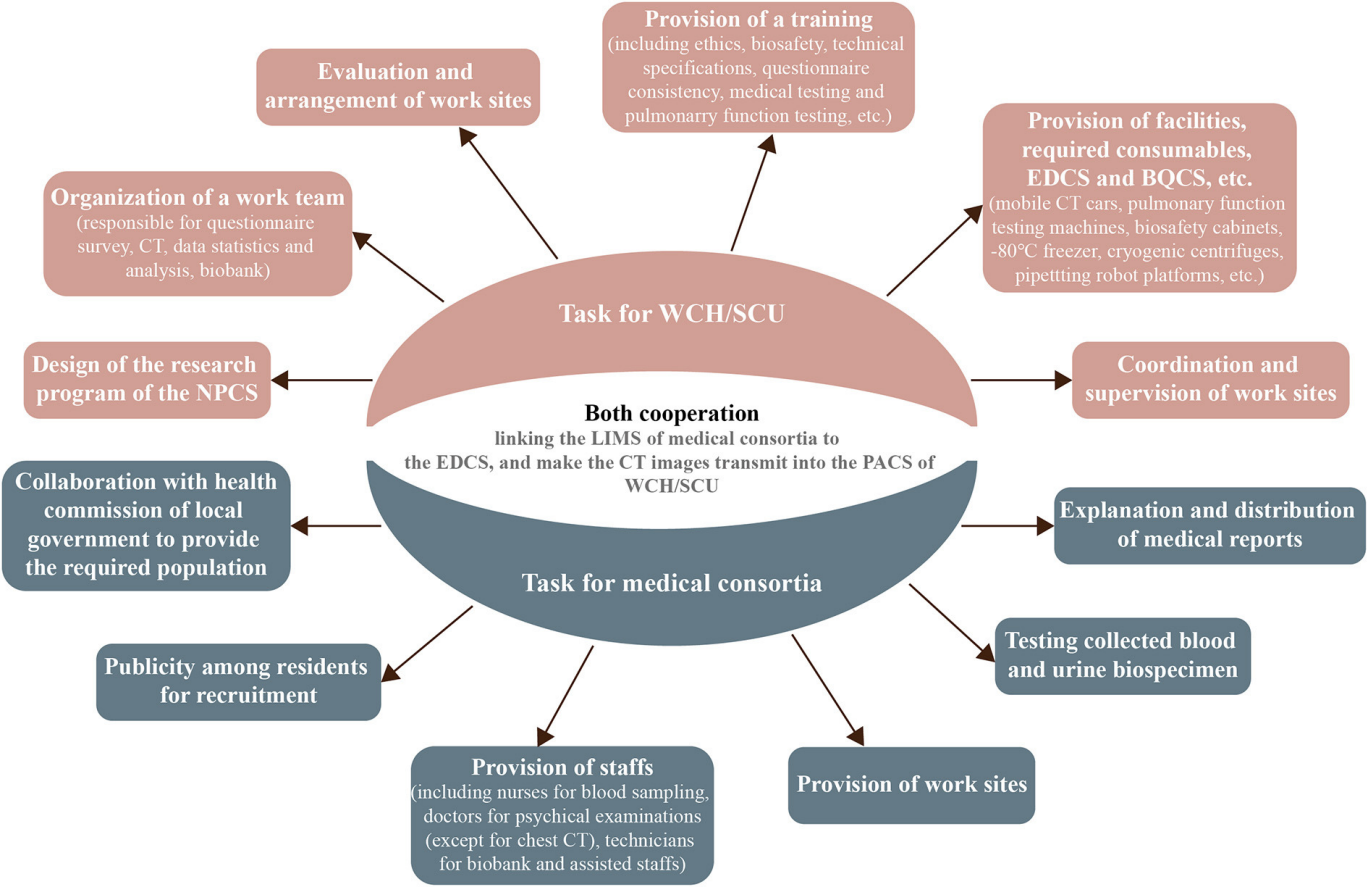
Task for WCH/SCU and medical consortia. WCH/SCU, West China Hospital of Sichuan University; NPCS, natural population cohort study of west China Hospital of Sichuan University; EDCS, electronic data capture system; BQCS, biospecimen quality control system; LIMS, laboratory information management system; PACS, picture archiving and communication system.

The assistance of the local government's health commission, where the 11 local centers are located, is of utmost importance. Moreover, effective cohort formation requires open communication and task delegation with a local medical consortium (Figure 2). Before carrying out designated NPCS programs at each site of the local medical consortia, the WCH/SCU will form a cohort research team comprising principal investigators, research assistants, medical technicians, and medical students to establish research protocols, equipment usage and maintenance, and information system management. After obtaining approval, the cohort research team will discuss detailed working plans with members of the local health commission and medical consortia. Thereafter, the cohort research team will invite experts from the WCH/SCU to provide systemic training for members who implement the NPCS programs. The NPCS programs will be advertised for participant enrolment by the local health commission and medical consortia. The local medical consortia will provide the working location, perform laboratory tests, and interpret the final medical examination report.

### Plan of information management

The information management of the NPCS consists of five main applications:

- The Electronic Data Capture System (EDCS) manages the information of questionnaires and parts of the health examination (height, weight, waist circumference, blood pressure, pulse, and pulmonary function).
- The Picture Archiving and Communication System (PACS) manages CT images and reports.
- The Laboratory Information Management System (LIMS) of medical consortia manages the biological parameters of the blood and urine.
- The Biospecimen Quality Control System (BQCS) collects key pre-analytical variables during sampling, processing, temporary storage, and biospecimen shipment.
- The Biobank Information Management System (BIMS) assembles the information for every biospecimen: the final storage location, pre-analytical variables in BQCS, the temperature of temporary storage and shipment, and long-term storage. In addition, it manages the banking/distribution, quality inspection data, feedback after use, and destruction records of biospecimens.

**Figure 3:**
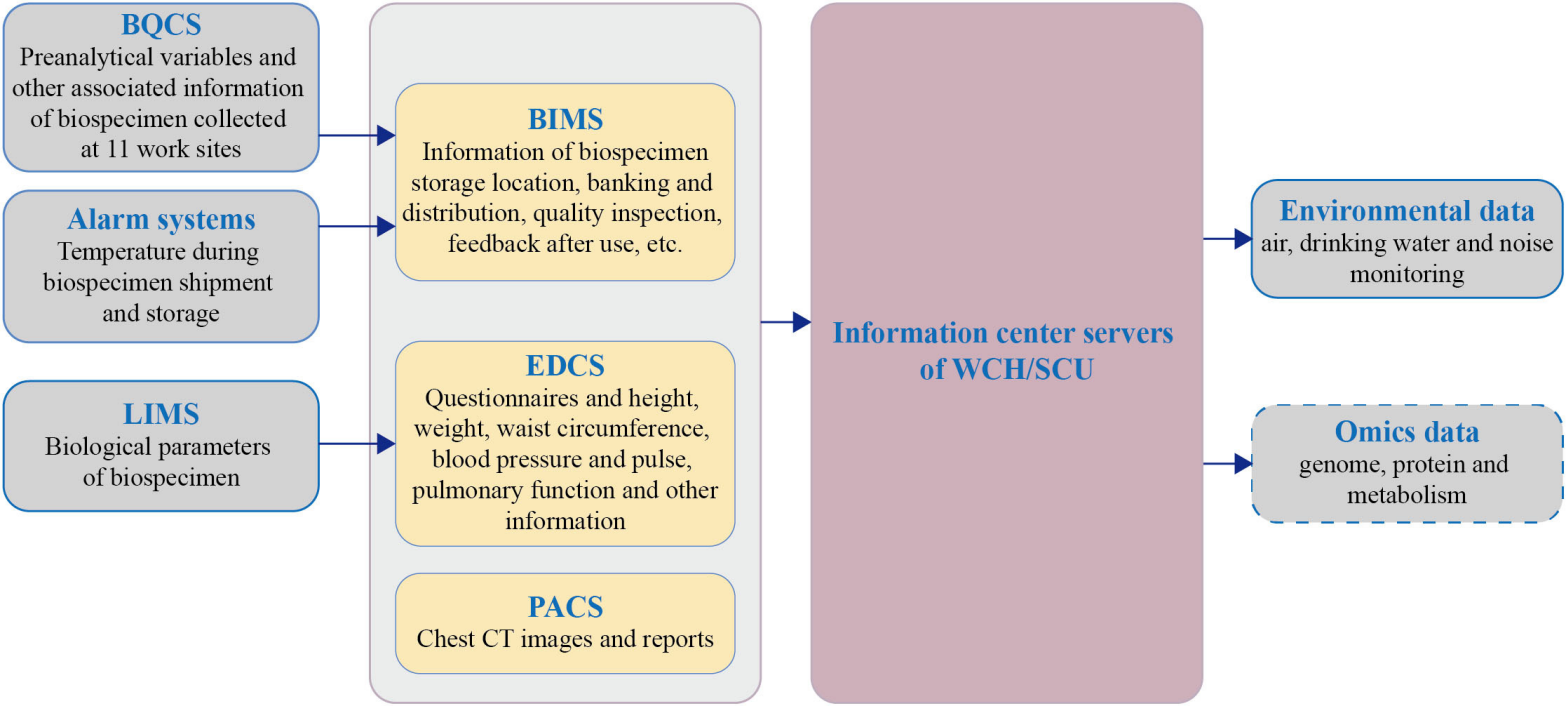
Flow diagram of information management for NPCS. BQCS, biospecimen quality control system; LIMS, laboratory information management system; BIMS, Biobank information management system; EDCS, electronic data capture system; PACS, picture archiving and communication system; WCH/SCU, West China Hospital of Sichuan University.

EDCS and PACS; LIMS; and BQCS and BIMS will be managed by staff responsible for health examination; medical laboratory staff; and staff from West China Biobanks, respectively. Eventually, information is aggregated into EDCS, PACS, and BIMS, and this information is then fused with local environmental data and uploaded to the information center of WCH/SCU for backup (Figure 3).

### Quality assurance and quality control

All quality assurance and quality control procedures adhered to the “DAIDS Guidelines for Good Clinical Laboratory Practice Standards” (Effective Date: 08/16/21) (28). To ensure the successful implementation of the NPCS, quality assurance procedures were established through rigorous methodological verification with preliminary experiments. Homogeneous training and SOPs are provided to the working staff responsible for questionnaire taking, health examination, blood withdrawal, sample processing, and any relevant procedures by experts of the WCH/SCU. The WCH/SCU will send medical technicians to local medical consortia for chest CT scans, and experts in the WCH/SCU will analyze the images. To ensure the homogeneity and quality of biospecimens collected from different local medical consortia, the West China Biobanks will send rotated staff under rigorous SOP training to each site for sample processing and storage. The daily checks are performed or repeated. In addition, periodic sampling inspection is conducted to ensure the storage condition of the biospecimens. Moreover, the questionnaire process, which is prone to deviation, could be corrected by re-examining the recorded questionnaire.

### Biospecimen collection, time interval, and transfer temperature

To qualify as approved sites for processing biospecimens, collaborating laboratories must meet the BSL-2 requirements or its latest standards to reduce the total risk to staff members. In principle, all participants should have their biosample donation before evaluated using a health questionnaire whenever possible, the total biosafety risk is minimal.

On the day of sample collection, fasting participants are encouraged to arrive at the local medical consortia between 7:50 and 10:30 a.m. Upon arrival, participants must complete the following steps according to the predefined schedule to donate their biospecimens (Table 1).

- Voluntarily sign an informed consent document.
- Registers using identity cards and two sets of barcodes are generated for the Biobank and the medical laboratory, respectively.
- Staff will attach the barcodes to vacutainers and sterile urine cups.
- The medical staff collect 20.5 mL of fasting blood into vacutainers. Vacutainers are gently inverted 10 times to allow anticoagulants or coagulants to mix thoroughly with blood. The barcodes for the Biobank are scanned in the BQCS to record the sampling time. Finally, vacutainers are placed upright on the racks.
- The participants deposit 50 mL urine samples into the urinary cup.

A 2–8°C freezer is available at the sampling site for temporary storage to reduce the period that blood is stored at room temperature. Technicians carry a temperature-controlled box to collect blood approximately every 30 min, whereas commercial couriers transport blood samples from health centers in villages or towns over time. During transport, these samples are stored at 2–8°C.

Once the biospecimen arrives at the processing facilities, the vacutainers are inspected (vacutainer number, barcode readability), and the end of transportation time is recorded in the BQCS. The collected blood and urine are then taken to the Biobank and medical testing department immediately upon arrival.

An approach combining automation and manual operation is implemented to aliquot the blood biospecimens. Two pipetting robot platforms (Integra Assist Plus, Sweden) and four cryogenic centrifuges are used to ensure the continuity of sample aliquoting. These solutions can cope with an average of 250 participants per day by up to eight technicians. They guarantee that LH plasma, SST serum, EDTA plasma, and buffy coat can be cryopreserved within 4 and 6 h, respectively, after venipuncture. The entire aliquoting operation is performed at room temperature, and the obtained aliquots are described (Table 2).

**Table 2:** Aliquoting and storage scheme.

**Type of biospecimen**	**Derivative**	**Aliquoting scheme**	**Cryotube**	**Aliquots for long-term storage**	
				**−80**°**C**	**Gas-Phase liquid nitrogen**
EDTA (9 mL)	Plasma	16 × 0.25 mL	Micronic 0.75 mL	8	8
	Buffy coat	4 × 0.50 mL	Micronic 0.75 mL	2	2
LH (3 mL)	Plasma	4 × 0.25 mL	Micronic 0.75 mL	2	2
SST (3 mL)	Serum	4 × 0.25 mL	Micronic 0.75 mL	2	2

EDTA, ethylene diamine tetraacetic acid; LH, lithium heparin; SST, serum separator tube.

Aliquoting is performed in the following steps. Blood biospecimens per vacutainer type are sorted manually, and barcodes are scanned into BQCS. The centrifugal conditions of each type of vacutainer are pre-set in the BQCS (the LH vacutainers are centrifuged at 1,500 × g for 10 min at 4°C. The SST vacutainers are centrifuged at 1,500 × g for 10 min at 4°C after standing for 25–30 min. The EDTA vacutainer centrifugation was divided into two steps: first, obtaining a buffy coat at 600 g for 10 min at 4°C; second, obtaining purified plasma at 1,500 g for 10 min at 4°C). The starting time of centrifugation is recorded in the BQCS, and the ending time is automatically generated. Then, the barcodes are scanned again to record the time of aliquot-starting in the BQCS. Aliquots of SST serum and LH plasma vacutainers are performed on pipetting robot platforms that suit the format of Cryoboxes and 2D barcoded cryotubes defined by the Society for Biomolecular Screening. The EDTA plasma is transferred to sterilized tubes manually for another centrifugation, and the resulting buffy coat is transferred to another sterilized tube. Both EDTA-derived fractions (plasma and buffy coat) are aliquoted onto pipetting robot platforms. The relevant operations are conducted in biosafety cabinets. The time of the aliquot ending is also recorded in the BQCS. Moreover, the barcodes of Cryoboxes and cryotubes are scanned into the BQCS to compare them with the pre-registered barcodes to ensure correct information. Finally, the Cryoboxes are placed in a monitored −80°C freezer for temporary storage, and the storage time is recorded in the BQCS; if not, they are placed in a 2–8°C freezer.

Notably, aliquots of each fraction are divided into two parts and fixed in the same position on the two Cryoboxes, aiming to improve management effectiveness during aliquoting and post-work.

### Biospecimen shipment to storage site

Select the Cryoboxes to be shipped in the BQCS, generate a shipment list, then compare the list information with the Cryobox barcode one by one. The Cryoboxes will be packed by a commercial courier if the information is correct. GPS and temperature probes are used separately to monitor the shipment process and temperature changes. Information on Cryoboxes and biospecimen types are carefully marked on each package. Finally, the biospecimen and a table for shipment are transferred to West China Biobanks by a commercial courier (−65 to −82°C) according to requirements specific to the project. The shipment starting and ending times are recorded in the BQCS.

### Biospecimen verification before long-term storage

Cryoboxes are assigned a storage location in BIMS in accordance with an electronic biospecimen shipment table submitted beforehand. Check the condition and shipment temperature of the biospecimens and whether the packages are broken when the biospecimens arrive at the West China Biobanks. A liquid nitrogen transfer truck is used to ensure that the biospecimens are in a low-temperature state before storage, and the number of biospecimens in each Cryobox is checked. Finally, two equivalent biospecimen aliquots are stored in gas-phase liquid nitrogen systems and −80°C freezers according to backup requirements. The biospecimen shipment table is signed and stored when the biospecimen information has been verified.

Two independent alarm systems are used to monitor −80°C freezers and a gas-phase liquid nitrogen system, which can be viewed through a mobile phone with personnel on duty daily. Empty gas-phase liquid nitrogen tanks are used for emergency transfers or planned maintenance.

### Access to resources

Research teams from the WCH/SCU or its network of medical consortia are given priority access to the biospecimens, and data collected by the NPCS. Applicants who wish to use the biospecimens and/or information are asked to draft a scientific protocol for their project to specify research objectives, methods, expected results, and requests (and reasons for their use). The NPCS also encourages national and international collaborators or industries as external users to apply biospecimens once a detailed agreement is reached. The protocols are then reviewed by the Scientific Committee of the WCH/SCU, and where applicable, by the ethics committee. For the approved projects, the requested information and the biospecimens are obtained from the information center, and from West China Biobanks, respectively. Currently, the resources are not open for external users because the NPCS project is in a clinical data tidy-up and genetic sequencing stage and is not ready for analysis.

### Bioinformatics analysis

Integrating multi-omics data to deeply explore the biological regulatory mechanisms at the transcriptional and post-transcriptional levels, as well as their relationships with other phenotypes such as diseases, are the common strategy to interpret genome-wide association studies (GWAS) signals. Given the detailed phenotype and the genotype from the NPCS cohort, GWAS can be performed for the quantitative traits or disease-control comparison. Additionally, with extensive multi-omics data, such as epigenetic and transcriptomic data, collected from the NPCS participants, we can implement an integrated analysis framework, including GWAS, PheWAS, expression quantitative trait loci (eQTL), burden analyses, and multi-level network regression to explore gene-phenotype correlation, genetic and environmental modification (26, 27, 29, 30), facilitating the exploration of the mechanism of coding or non-coding variants in diseases.

## Discussion

Several large-scale cohort studies focusing on the health of a natural population or specific group have been undertaken outside China in the past, and these studies have considerably increased our understanding of human diseases (31–37). Nonetheless, Chinese culture and lifestyle are distinct from those in other countries (2, 38). Consequently, it is essential to conduct natural cohort studies locally to account for these differences.

In 2001, the Ministry of Science and Technology of China initiated the National Infrastructure of Chinese Genetic Resources (NICGR), which is an important component of the National Science and Technology Infrastructure Program. After setting up the NICGR framework, China formally established several significant natural cohort studies based on local populations, which significantly increased our understanding of the pathophysiology of a local theme. For example, the Taizhou Longitudinal Study uses residents (>100,000 people) of Taizhou City, Jiangsu Province, as a framework to study the relationship between genes, environment, and their interactions with major chronic diseases (39). The China Kadoorie Biobank studies risk factors affecting the health of the Chinese population regarding genetics, environment, and lifestyle, using 500,000 individuals from 10 out of 34 of China's provincial administrative regions (40). While the NPCS likewise focuses on chronic disease research, its design is geographically specialized, considering many ethnic minorities, diverse lifestyles and religions, and high altitude, as well as plain, urban, and rural populations. Furthermore, within the framework of local medical consortia, the NPCS Biobank has deep clinical phenotypic, biochemical, and imaging data that are tightly linked with subsequently generated omics data for precision medicine research. It can not only serve as a valuable resource for research on major chronic and endemic diseases in Sichuan Province, but also as a representative of southwest population characteristics to enhance the creation of China's comprehensive health management policies.

More than 10 clinical departments of the WCH/SCU are involved in the ongoing project. It is anticipated that ~20 sub-studies on respiratory, cardiovascular, neurologic, digestive, geriatric, and other diseases will be based on NPCS. We hope to enlist the support of the Sichuan government to enable further data integration with data from the Centers for Disease Control and Prevention, Cancer Follow-up Registration Platform, and Healthcare Security Information Platform to ensure the stability of the cohort and the accuracy of the data gathered.

The NPCS is conducted in-depth cooperation with the medical consortia of the WCH/SCU to establish a multi-source, highly representative and long-term follow-up community cohort, and then through multi-dimensional data analysis to reveal the health status of Sichuan residents. At present, research on factors affecting sleep, rheumatoid arthritis, depression and anxiety, and lung nodule management have yielded a series of publications based on the NPCS. Of course, the current cohort is relatively small, and it may be difficult to study disease-predisposing genes in a short period. Moreover, there is population loss due to migrant workers, and minority people are hesitant to participate the project because of their living habits and religious beliefs, so this study cannot fully reflect the full picture of the local population. In addition, the investigation of specific diseases may be limited without collecting many types of biospecimens. Limited by the participants' educational level, the correctness of the questionnaire data in the plateau area must be carefully evaluated.

Overall, this paper offers a template for working with local medical consortia and guidance on creating a top-notch Biobank in local communities. The project will increase participants' awareness of their health, enhance their quality of life, and lessen the burden of disease. The capacity of medical consortia for research will increase due to the growth in interdisciplinary collaboration. In addition, the project has gained significant expertise in conducting extensive epidemiological investigations in urban and rural China. We hope that through the use of resources, the NPCS can promote the development of precision medicine, provide suitable participants for testing novel drugs and devices, cultivate interdisciplinary professionals, and ultimately improve the health status of Sichuan residents.

## Author contributions

LC and WH conceptualized the review and obtained funding. PF drafted the manuscript. PF, SZ, WW, ZY, WT, SL, CZ, DH, XZ, ZT, YW, and FL designed and implemented the NPCS Biobank. WH critically revised the manuscript. All authors have read and approved the manuscript before submission.

## Funding

This work was supported by the China National Key Research and Development Program (Grant No. 2020AAA0105005), Key Research and Development Projects of Sichuan Science and Technology Program (awarded to WH), National Clinical Research Center for Geriatrics (Z20201003), and Cluster Construction of Chinese Human Genetic Resources Biobanks in West China (2016YFC1201705).

## Conflict of interest

The authors declare that the research was conducted in the absence of any commercial or financial relationships that could be construed as a potential conflict of interest.

## Publisher's note

All claims expressed in this article are solely those of the authors and do not necessarily represent those of their affiliated organizations, or those of the publisher, the editors and the reviewers. Any product that may be evaluated in this article, or claim that may be made by its manufacturer, is not guaranteed or endorsed by the publisher.
