# Supplementary material for: The design and implementation of natural population cohort study Biobank: A multiple-center project cooperation with medical consortia in Southwest China
Source: Front Public Health. 2022 Dec 1;10:996169. doi: 10.3389/fpubh.2022.996169 (PMC9751194; doi:10.3389/fpubh.2022.996169)
Supplement: Supplementary file 1 [file Data_Sheet_1.pdf]

Supplementary Material

1 Supplementary Tables

Supplementary Table 1. Guidelines for chronic diseases management in China

Name	Time released	Affiliation
Guidelines for Diagnosis and Treatment of Primary Liver Cancer in China	2022	National Health Commission of the People's Republic of China
Guidelines for Clinical Diagnosis and Treatment of Lung cancer in China	2022	Chinese Medical Association
Guidelines for the Diagnosis and Management of Chronic Obstructive Pulmonary Disease in China	2021	Chinese Medical Association
Guidelines for Stroke Prevention and Treatment in China	2021	National Health Commission of the People's Republic of China
Precautionary Guidelines for Cardiovascular Diseases in China	2017	Chinese Medical Association

Supplementary Table 2. Exemplars of excellent international cohort studies and characteristic factors

Cohort name	Regions	Enroll start	Enrolled	Age of participants	Questionnaire and/or interview	Physical measures	Bio specimens	Genomics data	Proteomics data	Metabolomics data	Environmental data	Data sharing	Years of follow up	Main research directions based on disease	References
UK Biobank	England, Scotland, Wales	2006	500,000	40-69	YES	YES	YES	YES	YES	YES	YES	YES	/	Cancer, cardiovascular disease, stroke, diabetes, and dementia, etc.	[10]
Framingham Heart Study (Original cohort)	USA	1948	5,209	28-74	YES	YES	YES	NO	NO	NO	YES	YES	65	Cardiovascular disease	[11-13]
Framingham Heart Study (Offspring cohort)		1971	5,124	5-70	YES	YES	YES	YES	YES	YES	YES	YES	43		
Framingham Heart Study (Omni Cohort)		1994	507	27-78	YES	YES	YES	NO	NO	NO	YES	YES	≈15-20		
Framingham Heart Study (Third Generation cohort)		2002	4,095	19-72	YES	YES	YES	YES	YES	YES	YES	YES	≈10		
Framingham Heart Study (New offspring Spouse cohort)		2003	103	47-85	YES	YES	YES	NO	NO	NO	YES	YES	≈10		
Framingham Heart Study (Second generation omni cohort)		2003	410	20-80	YES	YES	YES	NO	NO	NO	YES	YES	≈10		
Nurses' Health Study (NHS I)	USA	1976	121700 (female)	30-55	YES	NO	YES	YES	YES	YES	YES	YES	Lifetime	Cancer, cardiovascular disease, postmenopausal	[14-16]

Nurses' Health Study (NHS II)		1989	116000 (female)	25-42	YES	NO	YES	YES	YES	YES	YES	YES	Lifetime	obesity, healthy aging, and psychosocial issues of aging, etc.	
Nurses' Health Study (NHS III)	USA, Canada	2010 (be rolling)	100,000 (female and male)	20-46	YES	NO	YES	YES	YES	YES	YES	YES	Lifetime		
Canadian Longitudinal Study on Aging	Canada	2010	50,000	45-85	YES	YES	YES	YES	YES	YES	YES	YES	>18	Healthy aging	[17]
Lifelines	Netherlands	2006	167,000	0-65+	YES	YES	YES	YES	NO	NO	YES	YES	>30	Healthy aging	[18-20]
European Prospective Investigation into Cancer and Nutrition study	France, Germany, Greece, Italy, Netherlands, Spain, UK, Sweden, Denmark, and Norway	1992	521,000	35-70 (mostly)	YES	YES	YES	YES	YES	YES	YES	YES	≈15	Cancer, cardiovascular disease, type 2 diabetes, mortality, and healthy ageing	[21-22]
German National Cohort	Germany	2014	200,000	20-69	YES	YES	YES	YES	YES	YES	YES	YES	25-30	Cancer, cardiovascular disease, diabetes, nervous system, rheumatic, or degrading diseases of the musculoskeletal system	[23]

Japan Public Health Center-based Prospective Study	Japan	1990	140,420	40-69	YES	YES	YES	YES	/	/	YES	YES	Lifetime	Cancer, myocardial infarction, stroke, etc.	[24]
Korean Genome and Epidemiology Study (Ansan and Ansung study)	Korea	2001	10,030	40-69	YES	YES	YES	YES	NO	NO	YES	YES	17	Cardiovascular disease, type 2 diabetes, hypertension, metabolic syndrome, and obesity	[25]
Korean Genome and Epidemiology Study (cardiovascular disease association study)		2004	28,338	>40	YES	YES	YES	YES	NO	NO	YES	YES	10		
Korean Genome and Epidemiology Study (health examinees study)		2004	173,343	>40	YES	YES	YES	YES	NO	NO	YES	YES	10		
Korean Genome and Epidemiology Study (twin and family study)		2005	3,202	/	YES	YES	YES	YES	NO	NO	YES	YES	7		
Korean Genome and Epidemiology Study (emigrant study)	Japan, China	2005	3,563	/	YES	YES	YES	YES	NO	NO	YES	YES	6		
Korean Genome and Epidemiology Study (immigrant study)	Korea, Vietnam, Cambodia	2006	11,245	/	YES	YES	YES	YES	NO	NO	YES	YES	3		

Supplementary Table 3. Questionnaire used in baseline survey

Name________________________ID________________________Date________________________Age________________________

Part 1: Basic Information			
**No.**	**Item**	**Response option**	**Your answer**
A1	Degree of Education	1- Elementary school and below<br>2- Junior high school<br>3- Senior high school or Vocational high school<br>4- Junior or undergraduate college<br>5- Master degree or above	
A2	Marital Status	1- Married or cohabiting<br>2- Unmarried<br>3- Divorced<br>4- Separated<br>5- Widowed	
A3	Participated Medical insurance	1- Uninsured<br>2- Basic medical insurance system for urban workers<br>3- Basic medical insurance system for non-working urban residents<br>4- New type of rural cooperative medical care system<br>5- Cooperative medical insurance for urban and rural residents<br>6- Commercial insurance<br>7- Unclear<br>8- Other insurance	
A4	Is it equal or more than five years since your house was built?	1- Yes, and resided for more than one year<br>2- Yes, and resided for less than one year<br>3- No	
A5	Were you born prematurely?	1- Yes<br>2- No<br>3- Unclear	
A6	Are you twins or multiples?	1- No<br>2- Identical twins<br>3- Fraternal twins<br>4- Multiple births	

A7	Is there any consanguineous marriage among your close relatives within four generations (you, your parents, your grandparents, and your great-grandparents)?	1- Yes<br>2- No<br>3- Unclear	
A8	Did your biological parents raise you?	1- Yes<br>2- No	
A9	Did your parents have any of the following negative events before you turned 17?	1- Divorced<br>2- Pass away<br>3- Alcoholism<br>4- Drug<br>5- Traffic accident<br>6- Crime<br>7- Lay ill in bed<br>8- None	

Part 2: Life-Style Behavior

**No.**	**Item**	**Response option**	**Your answer**
B1	Have you been screened for COVID-19?	1- Yes<br>2- No	
B2	Career	1- Principal of state agencies/party-masses/enterprise/project units<br>2- Professional/technical personnel<br>3- Clerk<br>4- Commercial/service personnel<br>5- Production personnel of agriculture/forestry/animal husbandry/fishery/water conservancy industry<br>6- Production and transportation equipment operators/related personnel<br>7- Soldier<br>8- Student (Jump to B11)<br>9- Laid-off/retired personnel (Jump to B11)<br>10- Unemployed (Jump to B11)<br>11- Other(s)	

B3	What kind of transportation do you take when you go out?	1- Public traffic<br>2- Private Cars<br>3- Motorcycle or electric cars<br>4- Bicycle<br>5- Pedestrian<br>6- Telecommuting (Jump to B5)	
B4	How far are you from the company?		________km
B5	How long do you work per week on average?		________day
B6	How long do you work per day on average?		________h
B7	How about the work intensity?	1- Low<br>2- Moderate<br>3- High	
B8	Main types of work	1- Mental labor<br>2- Manual work<br>3- Both	
B9	Do you spend long periods of time sitting/standing at daily work?	1- Long-term standing<br>2- Long-term sitting/sedentary, for________h<br>3- Neither<br>4- Both	
B10	How long is your sedentary time in free hours every weekday on average?		________h
B11	How long is your sedentary time on every rest day on average?		________h
B12	How did you go upstairs/downstairs?	1- Walking<br>2- Elevator<br>3- Combination of walking and elevator<br>4- No need to go upstairs/downstairs	

B13	In the past 6 months, what physical activity have you participated in?	1- None (Jump to B16)<br>2- Jogging<br>3- Walking<br>4- Stroll<br>5- Gymnasium<br>6- Yoga<br>7- Swimming<br>8- Aerobics<br>9- Ball game<br>10- Bicycle ride<br>11- Other exercise(s)__________	
B14	In the past 6 months, how many times a week have you participated in the above exercises?	1- Less than once a week<br>2- Once or twice a week<br>3- 3 to 5 times a week<br>4- 6 or more times a week	
B15	How long does each exercise last on average?		Number of minutes______
B16	How often do you cook at home?	1- Every day/Nearly every day<br>2- Not at all/Seldom (Jump to B19)<br>3- ________times a week on average	
B17	What is the main fuel used for cooking?	1- Gas<br>2- Electricity<br>3- Coal<br>4- Firewood<br>5- Other fuel(s)__________	
B18	How often do you use a fume extractor for cooking at home?	1- Always<br>2- Seldom<br>3- Never	
B19	What was your smoking status in the last six months?	1- Smoking<br>2- Occasional smoking<br>3- Not smoking (Jump to B24)<br>4- Quit smoking (Jump to B22)	
B20	When did you start smoking?		____(age)
B21	How many cigarettes did you smoke on average per day in the last week?	1- _________(quantity of cigarettes)<br>2- Zero	

B22	When did you quit smoking?		____(age)
B23	The main reason for quitting smoking?	1- Morbid or sub-health state<br>2- Disease prevention<br>3- Economic aspects<br>4- Other reason(s)_________	
B24	Are you exposed to passive smoking?	1- No (Jump to B27)<br>2- 1 or 2 times per week<br>3- 3 to 5 times per week<br>4- 5 or more times per week	
B25	Average time of the exposure to passive smoking	1- 15-29 min<br>2- 30-59 min<br>3- ⩾ 60 min	
B26	Do family members or roommates living with you smoke?	1- No<br>2- Parents<br>3- Grandparents<br>4- Spouse<br>5- Offspring<br>6- Roommate<br>7- Other(s)_________	
B27	Did you ever drink alcohol?	1- Yes<br>2- No (Jump to B33)<br>3- Quit (Jump to B32）	
B28	When did you start drinking?		____(age)

The following questions are about the past year

B29	How often do you have a drink containing alcohol?	1- Monthly or less<br>2- 2 to 4 times a month<br>3- 2 to 3 times a week<br>4- 4 or more times a week
B30	How many drinks containing alcohol do you have on a typical day when you are drinking?	1- 1 or 2<br>2- 3 or 4<br>3- 5 or 6<br>4- 7 or 9<br>5- 10 or more

B31	How often do you have six or more drinks on one occasion?	1- Less than monthly<br>2- Monthly<br>3- Weekly<br>4- Daily or almost daily	
B32	When did you quit drinking?		_________(age)

Pittsburgh Sleep Quality Index (PSQI)

B33	During the past month, when have you usually gone to bed at night?		Usual bedtime__
B34	During the past month, how long (in minutes) has it usually take you to fall asleep each night?		Number of Minutes_______
B35	During the past month, when have you usually gotten up in the morning?		Usual getting up time_______
B36	During the past month, how many hours of actual sleep did you get at night (This may be different than the number of hours you spend in bed)?		Hours of sleep per night______

During the past month, how often have you had trouble sleeping because you...

B37	Cannot get to sleep within 30 minutes	1- Not during the last month<br>2- Less than once a week<br>3- Once or twice a week<br>4- Three or more times a week	
B38	Wake up in the middle of the night or early morning	1- Not during the last month<br>2- Less than once a week<br>3- Once or twice a week<br>4- Three or more times a week	
B39	Have to get up to use the bathroom	1- Not during the last month<br>2- Less than once a week<br>3- Once or twice a week<br>4- Three or more times a week	

B40	Cannot breathe comfortably	1- Not during the last month<br>2- Less than once a week<br>3- Once or twice a week<br>4- Three or more times a week
B41	Cough or snore loudly	1- Not during the last month<br>2- Less than once a week<br>3- Once or twice a week<br>4- Three or more times a week
B42	Feel too cold	1- Not during the last month<br>2- Less than once a week<br>3- Once or twice a week<br>4- Three or more times a week
B43	Feel too hot	1- Not during the last month<br>2- Less than once a week<br>3- Once or twice a week<br>4- Three or more times a week
B44	Had bad dreams	1- Not during the last month<br>2- Less than once a week<br>3- Once or twice a week<br>4- Three or more times a week
B45	Have pain	1- Not during the last month<br>2- Less than once a week<br>3- Once or twice a week<br>4- Three or more times a week
B46	Other reason(s), please describe_____________	1- Not during the last month<br>2- Less than once a week<br>3- Once or twice a week<br>4- Three or more times a week
B47	During the past month, how would you rate your sleep quality overall?	1- Superior/Very good<br>2- General/Fairly good<br>3- Inferior/Fairly bad<br>4- Poor/Very bad
B48	During the past month, how often have you taken medicine (prescribed or "over the counter") to help you sleep?	1- Not during the last month<br>2- Less than once a week<br>3- Once or twice a week<br>4- Three or more times a week
B49	During the past month, how often have you had trouble staying awake while driving, eating meals, or engaging in social activity?	1- Not during the last month<br>2- Less than once a week<br>3- Once or twice a week<br>4- Three or more times a week

B50	During the past month, how much of a problem has it been for you to keep up enough enthusiasm to get things done?	1- Not during the last month<br>2- Less than once a week<br>3- Once or twice a week<br>4- Three or more times a week	

Part 3: Sign and Symptom

No.	Item	Response option	Your answer
C1	During the two months, has your weight dropped significantly?	1- Yes<br>2- No (Jump to C3)	
C2	Weight loss		________kg
C3	Do you have the following symptoms?	1- Pant<br>2- Dyspnea<br>3- Hoarseness<br>4- Dysphagia<br>5- Chest tightness<br>6- None	
C4	Do you have the history of occupational exposure to radon, asbestos, arsenic, dust and lampblack?	1- Radon, for_________years<br>2- Asbestos, for_________years<br>3- Arsenic, for_________years<br>4- Dust, for__________years<br>5- Lampblack, for_________years<br>6- None	
C5	Have you been diagnosed with intrathoracic sarcoidosis by your doctor before?	1- Yes, pulmonary nodules were first detected at___________<br>2- No (Jump to C7)	
C6	Pulmonary nodules were first detected by	1- Health examination<br>2- Clinical examination	
C7	Have you been diagnosed COPD by your doctor?	1- Yes, _________(age at diagnosis)<br>2- No<br>3- Unclear	
C8	Do you currently take more than five medications orally?	1- Yes<br>2- No	

C9	Do you have frequent dental ulcers?	1- Yes<br>2- No
C10	Are you allergic to sunlight?	1- Yes<br>2- No
C11	Does your skin appear pale and bruised after exposure to cold?	1- Yes<br>2- No
C12	Do you have joint pain?	1- No<br>2- Distal interphalangeal joint<br>3- Proximal interphalangeal joint<br>4- Metacarpophalangeal joint<br>5- Wrist joints<br>6- Elbow joint<br>7- Shoulder joint<br>8- Temporo-mandibular Joint<br>9- Hip joint<br>10- Knee joint<br>11- Ankle joint<br>12- Metatarsophalangeal joint<br>13- Sternoclavicular joint<br>14- Sacroiliac joint<br>15- Calcaneal-talar joint<br>16- Other(s)__________
C13	During the past month, have you had repetitive knee joint pain?	1- Yes<br>2- No
C14	Does the knee joint produce bone crepitus when moving?	1- Yes<br>2- No
C15	Have you ever had a knee injury?	1- Yes<br>2- No
C16	During the past year, have you repeatedly experienced one of the following situations?	1- No<br>2- Joint pain appeared in both hands, which is worst in the morning with stiffness (⩾30 min) and gets better with activity<br>3- Morning stiffness (<30 min) in knee
C17	Have you been diagnosed with migraine by your doctor before?	1- Yes<br>2- No

C18	Do you had headaches when you're young (at age of 10-50), and which limit your ability to work, study or enjoy life?	1- Yes<br>2- No
C19	Do you feel nauseated or sick to your stomach with headaches?	1- Yes<br>2- No
C20	Does the light bother you?	1- Yes<br>2- No
C21	Have you been diagnosed with Parkinson's disease (PD) by your doctor?	1- Yes, __________(age at diagnosis), ___________(hospital)<br>2- No (Jump to C23)
C22	What type of medications are you taking currently?	1- No-drug treatment<br>2- Unclear<br>3- Madopar<br>4- Pramipexole<br>5- Entacapone<br>6- Trihexyphenidyl<br>7- Amantadine<br>8- Sinemet<br>9- Trastal<br>10- Eldepryl<br>11- Rasagiline Mesylate Tablets<br>12- Vitamin B6
C23	Have you been diagnosed with cerebral infarction by your doctor?	1- Yes, __________(age at diagnosis), ___________(hospital)<br>2- No (Jump to C25)
C24	What type of medications are you taking currently?	1- No-drug treatment<br>2- Unclear<br>3- Aspirin<br>4- Clopidogrel<br>5- Warfarin<br>6- Pradaxa<br>7- Rivaroxaban<br>8- Atorvastatin<br>9- Simvastatin
C25	Have you been diagnosed with cerebral hemorrhage by your doctor?	1- Yes, __________(age at diagnosis), ___________(hospital)<br>2- No (Jump to C27)

C26	What type of medications are you taking currently?	1- No-drug treatment<br>2- Unclear<br>3- Aspirin<br>4- Clopidogrel<br>5- Warfarin<br>6- Pradaxa<br>7- Rivaroxaban<br>8- Atorvastatin<br>9- Simvastatin
C27	Have you been diagnosed with epilepsy by your doctor?	1- Yes, __________(age at diagnosis), __________(hospital)<br>2- No (Jump to C29)
C28	What type of medications are you taking currently?	1- No-drug treatment<br>2- Unclear<br>3- Sodium valproate<br>4- Levetiracetam<br>5- Topiramate<br>6- Carbamazepine<br>7- Oxcarbazepine<br>8- Lamotrigine<br>9- Phenytoin sodium<br>10- Phenobarbital<br>11- Clonazepam<br>12- Lacosamide
C29	Have you been diagnosed with other neurological disorders by your doctor?	1- Yes, _________(age at diagnosis), _________(hospital)<br>2- No (Jump to C31)
C30	Have you taken any medicine?	1- Yes, _________(drug name)<br>2- No
C31	Have you been diagnosed with lithiasis by your doctor?	1- No<br>2- Unclear<br>3- Lithiasis in urinary system<br>4- Hepatobiliary lithiasis<br>5- Other(s)___________
C32	During the past year, have you ever leaked urine involuntarily?	1- Yes<br>2- No<br>3- Refuse to answer<br>4- Unclear

C33	How many times do you get up to urinate at night from falling asleep to waking up?	1- Yes, __________times per night<br>2- No<br>3- Refuse to answer<br>4- Unclear
C34	During the past year, have you been able to urinate smoothly (male only)?	1- Yes<br>2- No<br>3- Refuse to answer<br>4- Unclear
C35	How many times do you defecate in a day?	1- Less than once a day<br>2- Once a day<br>3- Twice a day<br>4- More than twice a day
C36	When do you usually defecate?	1- Morning<br>2- Forenoon<br>3- None<br>4- Afternoon<br>5- Night<br>6- Random
C37	Have you been diagnosed with hypertension by your doctor?	1- Yes, _________(age at diagnosis), __________(hospital)<br>2- No
C38	Have you been diagnosed with hyperglycemia by your doctor?	1- No<br>2- Diabetes mellitus, ___________(age at diagnosis)<br>3- Prediabetes, ____________(age at diagnosis)<br>4- Unknown/other type(s), ___________(age at diagnosis)
C39	Have you been diagnosed with a tumor by your doctor?	1- No (Jump to C41)<br>2- Benign<br>3- Malignant<br>4- Both benign and malignant<br>5- Unclear

C40	Please select your specific tumor type	1- Breast cancer<br>2- Lung cancer<br>3- Ovarian cancer<br>4- Metrocarcinoma<br>5- Brain tumor<br>6- Gastric carcinoma<br>7- Liver cancer<br>8- Colorectal cancer<br>9- Esophagus cancer<br>10- Bladder cancer<br>11- Pancreatic cancer<br>12- Hysteromyoma<br>13- Hepatic cyst<br>14- Prostatic cancer<br>15- Ovarian cyst<br>16- Other tumor type(s),____________
C41	Have you been diagnosed with the following diseases by your doctor (thyroid-associated diseases are not included)?	1- No<br>2- CHD (myocardial infarction/angina)<br>3- Systemic lupus erythematosus<br>4- Osteoporosis<br>5- Kidney-related diseases<br>6- Depression<br>7- Schizophrenia<br>8- Hyperlipidemia<br>9- Pulmonary heart disease<br>10- Chronic heart failure<br>11- Chronic hepatitis virus<br>12- Fatty liver disease<br>13- Liver cirrhosis<br>14- Gout and hyperuricemia<br>15- Interstitial pneumonia<br>16- Chronic bronchitis<br>17- Emphysema<br>18- Asthma<br>19- Tuberculosis<br>20- Pneumonoconiosis<br>21- Rheumatoid Arthritis<br>22- Knee osteoarthritis<br>23- Diabetic retinopathy<br>24- diabetic foot<br>25- Diabetic foot amputations

		26- Diabetic blindness<br>27- History of benign breast disease<br>28- Coronary heart disease<br>29- Heart failure<br>30- Artery disease (arteriosclerosis/plaque/arteriostenosis)<br>31- Atrial fibrillation<br>32- Brain trauma<br>33- Viral encephalitis<br>34- Benign prostatic hyperplasia<br>35- Acute pancreatitis<br>36- Other pancreatic diseases<br>37- Scleroderma<br>38- Dermatomyositis<br>39- Sicca syndrome<br>40- Gastric ulcer<br>41- Functional dyspepsia<br>42- Cholangiectasis<br>43- Other disease(s),___________	
C42	Have you had any type of surgery?	1- Yes<br>2- No (Jump to C46)	
C43	How old were you when you had your first operation?		____(age)
C44	What is the reason for your operation?		____(reason)
C45	What is your type of surgery?	1- Open Surgery<br>2- Minimally invasive surgery<br>3- Other type(s)________	
C46	Have you ever had a fracture?	1- Yes<br>2- No (Jump to C49)	
C47	Did your fracture occur in a violent situation (road accident injury /drifting-down injury/bruise injury caused by heavy object etc.)?	1- Yes<br>2- No	

C48	Where is your fracture location?	1- Spine<br>2- Hip/Femur<br>3- Forearm<br>4- Upper arm<br>5- Pelvis<br>6- Ankle<br>7- Rib<br>8- Other body part(s),_________
C49	Have you been diagnosed with thyroid diseases by your doctor?	1- Hyperthyroidism<br>2- Hypothyroidism<br>3- Graves disease<br>4- Hashimoto's thyroiditis<br>5- Thyroid tumor<br>6- Thyroid nodule<br>7- No
C50	Have your direct lineal consanguinity in blood and full siblings within three generations been diagnosed with the following diseases by the doctor?	1- No<br>2- Unclear<br>3- Diabetes mellitus<br>4- Hyperlipidemia<br>5- Hypertension<br>6- Lung cancer<br>7- Prostatic cancer<br>8- Other malignant tumor(s)<br>9- Hyperthyroidism<br>10- Hypothyroidism<br>11- Graves disease<br>12- Hashimoto's thyroiditis<br>13- Pancreatitis<br>14- Knee Arthritis<br>15- Ankylosing Spondylitis<br>16- Systemic Lupus Erythematosus<br>17- Rheumatoid arthritis<br>18- Scleroderma<br>19- Dermatomyositis<br>20- Sicca syndrome<br>21- Polycystic kidney disease<br>22- Familial hypercholesterolemia<br>23- Hypertrophic cardiomyopathy<br>24- Dilated cardiomyopathy<br>25- Arrhythmogenic Right Ventricular Cardiomyopathy<br>26- Long QT syndrome

		27- Catecholaminergic polymorphic ventricular tachycardia<br>28- Brugada syndrome<br>29- Marfan syndrome<br>30- Liddle’s syndrome<br>31- Familial Aldosteronism<br>32- Glucocorticoid-remediable aldosteronism

Part 4: Female Relevant Information (Female only)

No.	Item	Response option	Your answer
D1	When did you first have a period?	1- First menstruation, _______(age)<br>2- No menstruation (Jump to D3), because of__________	
D2	How long does your period last?	1- < 3 days<br>2- 3-7 days<br>3- > 7 days<br>4- Irregular	
D3	How many children have you had?	1- None (Jump to D6)<br>2- One<br>3- ⩾ Two,_______(specific number)	
D4	When did you have your first child?		____(age)
D5	How long have you been breast-feeding?	1- 1-6 months<br>2- 7-12 months<br>3- 13-24 months<br>4- > 24 months<br>5- Not breast-feeding	
D6	Have you been diagnosed with pregnancy diseases by your doctor?	1- No<br>2- Gestational hypertension<br>3- Eclampsia<br>4- Gestational diabetes mellitus<br>5- Hyperthyroidism/hypothyroidism in pregnancy<br>6- Other disease(s),__________	

D7	Have you ever had an abortion?	1- No<br>2- Pathologic labor,__________(abortion times)<br>3- Family planning policy,_________(abortion times)<br>4- Other reason(s), __________(abortion times)	
D8	Have you had three or more unexplained miscarriages?	1- Yes<br>2- No	
D9	Are you menopausal?	1- Premenopause (Jump to E1)<br>2- Perimenopause (Jump to E1)<br>3- Postmenopause	
D10	When did you go through menopause?		____(age)
D11	Did you go through menopause naturally?	1- Yes<br>2- No	

Part 5: Diet

No.	Item	Response option	Your answer
E1	Are you living alone?	1- Yes<br>2- No	
E2	Are you eating alone?	1- Yes<br>2- No,_________(specific number)	
E3	What type of drinking water do you prefer?	1- Tap water<br>2- Bottled water<br>3- Well water<br>4- Purified water<br>5- Pure water<br>6- Bottled Mineral Water<br>7- Spring Water<br>8- Other type(s),_________	

E4	What kind of oil do you prefer?	1- Canola oil<br>2- Soybean oil<br>3- Peanut oil<br>4- Olive oil<br>5- Corn oil<br>6- Sesame oil<br>7- Animal oil<br>8- Other type(s),___________
E5	What is the taste preference of your daily diet?	1- Greasy<br>2- Spicy<br>3- Salty<br>4- Light<br>5- Sweet<br>6- Hot<br>7- Moderate
E6	What is the texture requirement of the food you eat?	1- Hard<br>2- Moderate<br>3- Soft
E7	What is the temperature requirement of the food you eat?	1- Hot<br>2- Warm<br>3- Cold
E8	How fast do you usually eat?	1- Slowly (> 45min)<br>2- Moderately<br>3- Fast (< 15min)
E9	Do you have a regular diet?	1- Yes<br>2- No (Jump to E11)
E10	When is your mealtime?	1- Breakfast,_________(specific meal time)<br>2- Lunch,_________(specific meal time)<br>3- Dinner,_________(specific meal time)<br>4- Extra meal 1,_________(specific meal time)<br>5- Extra meal 2,_________(specific meal time)
E11	What is the average frequency of your breakfast each week?	1- Home,_________(times)<br>2- Restaurant,_________(times)<br>3- Canteen,_________(times)<br>4- Take-away,_________(times)
E12	What is the average frequency of your lunch each week?	1- Home,_________(times)<br>2- Restaurant,_________(times)<br>3- Canteen,_________(times)<br>4- Take-away,_________(times)

E13	What is the average frequency of your lunch each week?	1- Home,_________(times)<br>2- Restaurant,_________(times)<br>3- Canteen,_________(times)<br>4- Take-away,_________(times)
E14	What is the average frequency of your lunch each week?	1- Home,_______(times)<br>2- Restaurant,_______(times)<br>3- Canteen,_______(times)<br>4- Take-away,_______(times)
E15	Please sort your meals according to food intake	1- Breakfast > Lunch > Dinner<br>2- Lunch > Breakfast > Dinner<br>3- Lunch > Dinner > Breakfast<br>4- Breakfast > Dinner > Lunch<br>5- Dinner > Lunch > Breakfast<br>6- Dinner > Breakfast > Lunch<br>7- Equal
E16	How often do you eat sweets each week?	1- Almost not<br>2- Seldom (Once or twice a week）<br>3- Always (three to five times a week)<br>4- Daily (five or more times a week)
E17	How often do you eat fried food each week?	1- Almost not<br>2- Seldom (Once or twice a week）<br>3- Always (three to five times a week)<br>4- Daily (five or more times a week)
E18	How often do you drink tea each week?	1- Almost not<br>2- Seldom (Once or twice a week）<br>3- Always (three to five times a week)<br>4- Daily (five or more times a week)
E19	How often do you drink coffee each week?	1- Almost not<br>2- Seldom (Once or twice a week）<br>3- Always (three to five times a week)<br>4- Daily (five or more times a week)
E20	In addition to your daily diet, do you regularly take vitamin or mineral supplements?	1- Yes<br>2- Irregular<br>3- No
E21	In addition to your daily diet, do you regularly take probiotic supplements?	1- Yes<br>2- Irregular<br>3- No

E22	In addition to your daily diet, do you regularly take other nutritional supplements (e.g. melatonin/protein powder/dietary fiber/meal replacement powder)?	1- Yes<br>2- Irregular<br>3- No	

Part 6: Psychological State

No.	Item	Response option	Your answer
**F1-Questionnaire 1**<br>(Please answer all questions carefully. This questionnaire asks people to rate themselves and select the corresponding option according to different circumstances. The higher scores represented the more agreeable with the point of view)			
F1-1	In uncertain times, I usually expect the best.	1- Strongly disagree<br>2- Disagree<br>3- Neutral<br>4- Agree<br>5- Strongly agree	
F1-2	If something can go wrong for me, it will.	1- Strongly disagree<br>2- Disagree<br>3- Neutral<br>4- Agree<br>5- Strongly agree	
F1-3	I always optimistic about my future.	1- Strongly disagree<br>2- Disagree<br>3- Neutral<br>4- Agree<br>5- Strongly agree	

F1-4	Things never work out the way I want them to.	1- Strongly disagree<br>2- Disagree<br>3- Neutral<br>4- Agree<br>5- Strongly agree
F1-5	I rarely count on good things happening to me.	1- Strongly disagree<br>2- Disagree<br>3- Neutral<br>4- Agree<br>5- Strongly agree
F1-6	In general, I prefer good things to bad things to happen to me.	1- Strongly disagree<br>2- Disagree<br>3- Neutral<br>4- Agree<br>5- Strongly agree

F2-Questionnaire 2

(Please answer all questions carefully. This questionnaire asks people to rate themselves and select the corresponding option according to different circumstances. The higher scores represented the more agreeable with the point of view)

F2-1	In most ways my life is close to my ideal.	1- Strongly disagree<br>2- Disagree<br>3- Slightly disagree<br>4- Neither agree nor disagree<br>5- Slightly agree<br>6- Agree<br>7- Strongly agree
F2-2	The conditions of my life are excellent.	1- Strongly disagree<br>2- Disagree<br>3- Slightly disagree<br>4- Neither agree nor disagree<br>5- Slightly agree<br>6- Agree<br>7- Strongly agree
F2-3	I am satisfied with my life.	1- Strongly disagree<br>2- Disagree<br>3- Slightly disagree<br>4- Neither agree nor disagree<br>5- Slightly agree

		6- Agree<br>7- Strongly agree
F2-4	So far I have gotten the important things I want in life.	1- Strongly disagree<br>2- Disagree<br>3- Slightly disagree<br>4- Neither agree nor disagree<br>5- Slightly agree<br>6- Agree<br>7- Strongly agree
F2-5	If I could live my life over, I would change almost nothing.	1- Strongly disagree<br>2- Disagree<br>3- Slightly disagree<br>4- Neither agree nor disagree<br>5- Slightly agree<br>6- Agree<br>7- Strongly agree

F3-UCLA Loneliness Scale (Simplified Version)

(Please read each section carefully. Larger number means higher frequency of occurrence.
This questionnaire asks people to rate themselves and select the corresponding option according to different circumstances)

F3-1	Lack companionship	1- Never<br>2- Sometimes<br>3- Always
F3-2	Feel left out	1- Never<br>2- Sometimes<br>3- Always
F3-3	Feel isolated from others	1- Never<br>2- Sometimes<br>3- Always

F4-Generalized Anxiety Disorder 7-item Scale (GAD7)

(Over the last 2 weeks, how often have you been bothered by any of the following problems?)

F4-1	Feeling nervous, anxious, or on edge	1- Not at all<br>2- Several days<br>3- More than1 week<br>4- Nearly every day

F4-2	Feeling hard to stop or control anxiety	1- Not at all<br>2- Several days<br>3- More than1 week<br>4- Nearly every day
F4-3	Excessive anxiety about different things	1- Not at all<br>2- Several days<br>3- More than1 week<br>4- Nearly every day
F4-4	Trouble relaxing	1- Not at all<br>2- Several days<br>3- More than1 week<br>4- Nearly every day
F4-5	Being so restless that it is hard to sit still	1- Not at all<br>2- Several days<br>3- More than1 week<br>4- Nearly every day
F4-6	Becoming easily annoyed or irritable	1- Not at all<br>2- Several days<br>3- More than1 week<br>4- Nearly every day
F4-7	Feeling afraid as if something awful might happen	1- Not at all<br>2- Several days<br>3- More than1 week<br>4- Nearly every day

F5-Patient Health Qustionnaire-9 (PHQ-9)

(Over the last 2 weeks, how often have you been bothered by any of the following problems?)

F5-1	Little interest or pleasure in doing things	1- Not at all<br>2- Several days<br>3- More than half the days<br>4- Nearly every day
F5-2	Feeling down, depressed, or hopeless	1- Not at all<br>2- Several days<br>3- More than half the days<br>4- Nearly every day
F5-3	Trouble falling or staying asleep, or sleeping too much	1- Not at all<br>2- Several days<br>3- More than half the days<br>4- Nearly every day

F5-4	Feeling tired or having little energy	1- Not at all<br>2- Several days<br>3- More than half the days<br>4- Nearly every day
F5-5	Poor appetite or overeating	1- Not at all<br>2- Several days<br>3- More than half the days<br>4- Nearly every day
F5-6	Feeling bad about yourself or that you are a failure or have let yourself or your family down	1- Not at all<br>2- Several days<br>3- More than half the days<br>4- Nearly every day
F5-7	Trouble concentrating on things, such as reading the newspaper or watching television	1- Not at all<br>2- Several days<br>3- More than half the days<br>4- Nearly every day
F5-8	Moving or speaking so slowly that other people could have noticed; Or the opposite—being so fidgety or restless that you have been moving around a lot more than usual	1- Not at all<br>2- Several days<br>3- More than half the days<br>4- Nearly every day
F5-9	Thoughts that you would be better off dead or of hurting yourself in some way	1- Not at all<br>2- Several days<br>3- More than half the days<br>4- Nearly every day
